# A Design of a Novel Silicon Photonics Sensor with Ultra-Large Free Spectral Range Based on a Directional Coupler-Assisted Racetrack Resonator (DCARR)

**DOI:** 10.3390/s23115332

**Published:** 2023-06-04

**Authors:** Osamah Alsalman, Iain Crowe

**Affiliations:** 1 Department of Electrical Engineering, College of Engineering, King Saud University, P.O. Box 800, Riyadh 11421, Saudi Arabia; 2 Department of Electrical and Electronic Engineering, Photon Science Institute, The University of Manchester, Manchester M13 9PL, UK

**Keywords:** micro-ring resonator, racetrack resonator (RR), directional coupler (DC), vernier effect, refractive index sensing

## Abstract

A novel refractive index-based sensor implemented within a silicon photonic integrated circuit (PIC) is reported. The design is based on a double-directional coupler (DC) integrated with a racetrack-type resonator (RR) to enhance the optical response to changes in the near-surface refractive index via the optical Vernier effect. Although this approach can give rise to an extremely large ‘envelope’ free spectral range (FSR*_Vernier_*), we restrict the design geometry to ensure this is within the traditional silicon PIC operating wavelength range of 1400–1700 nm. As a result, the exemplar double DC-assisted RR (DCARR) device demonstrated here, with FSR*_Vernier_* = 246 nm, has a spectral sensitivity *S_Vernier_* = 5 × 10^4^ nm/RIU.

## 1. Introduction

An evanescent wave is a type of electromagnetic wave that exists only in the immediate vicinity of a boundary between two media. Unlike propagating waves, which can travel indefinitely through a medium, evanescent waves decay exponentially as they move away from the boundary. This phenomenon can be both useful and parasitic in a variety of applications. In photonics, for example, it can be used to couple light into and out of optical fibres or waveguides or to transfer energy between different waveguides (i.e., directional couplers). In sensing applications, the interaction between evanescent fields and different molecules leads to perturbations in the effective index ($n_{eff}$) of the propagating light in a fibre or waveguide, which can be utilised to detect changes in the refractive index (RI) of a sample [1].

However, evanescent waves or evanescent coupling can also be parasitic in certain situations. In some cases, for example, evanescent coupling between adjacent waveguides can result in signal crosstalk, which can degrade device performance. Similarly, in some sensing applications, evanescent fields can result in unwanted cross-sensitivity to environmental variables.

Controlling evanescent waves and evanescent coupling is key to minimising their parasitic effects and maximising their usefulness. One approach to controlling evanescent waves and coupling is to engineer the geometry of the objects involved, such as the shape and size of the waveguides or fibres. This can help to minimise unwanted coupling and maximise the desired coupling. Another approach is to use materials with specific refractive indices, which can affect the strength and range of evanescent coupling.

Nowadays, many approaches employ evanescent waves for sensing purposes, such as surface plasmon resonance (SPR) [2], optical fibre [3] and integrated optical sensors. The waveguide-based structures enable much smaller size, more flexible and inexpensive sensors.

Integrated silicon rings or racetrack resonators, traditionally associated with optical filtering and switching in telecom applications, have emerged as key components for sensing in recent years. By utilising quasi-TE mode polarisation, conventional single RRs based on silicon-on-insulator (SOI), technology can attain bulk sensitivities of around 70 to 133 nm/RIU [4,5]. However, this is limited by the fact that the interaction between adsorbed molecules and the guided mode evanescent field is highly surface sensitive, with an interaction range of just a few hundred nanometres from the waveguide core. This may be overcome, at least in part, by using slotted, concentric- [6,7], cascaded- [8] or subwavelength-grating (SWG)-RRs [9] or by the integration of functional layers, capable of better adsorbing or chemically activating, surface-bound molecular analytes. In some cases, such approaches were also reported to provide a degree of selectivity [10,11,12].

**Figure 1:**
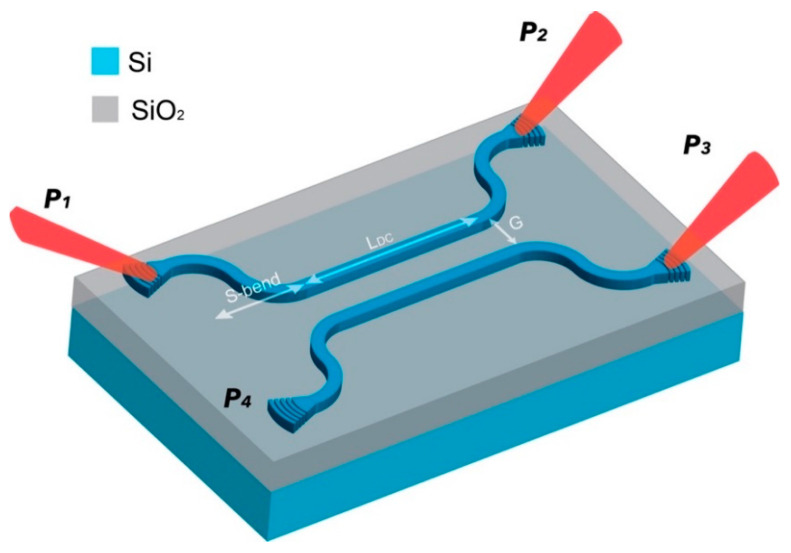
Schematic of a standard DC formed from two adjacent silicon waveguides (fabricated from a silicon-on-insulator (SOI) starting wafer), terminated in low loss ‘S-bends’ and Bragg-type grating couplers. Light is coupled into the grating coupler port, *P*_1_ and is split, via evanescent coupling over the parallel waveguide section, by a ratio determined by the coupling length, *L_DC_*_,_ with the corresponding output signal(s) collected at ports, *P*_2_ and *P*_3_.

Another key component in the silicon photonics ‘toolkit’ is the directional coupler (DC), which has also traditionally found application in, e.g., optical routing of signals for telecoms. DCs consist of two (typically parallel) waveguides in close enough proximity (a few hundred nm’s) such that the propagating optical mode in one of the guides may be evanescently coupled to the adjacent guide, re-routing all or part of the optical signal, over a broad wavelength range, Figure 1.

The electromagnetic waves can be coupled into and out of the DC via grating couplers. The coupled light at port *P*_1_ might be efficiently split between *P*_2_ and *P*_3_ or even entirely transferred to *P*_3_ depending on the coupling coefficient, *k*. This coefficient describes the ratio of the power splitting (i.e., coupling strength) between the parallel sections of the DC. It is determined by several parameters, including the waveguide material (index) and geometry, separation gap, G, length of the coupling region, *L_DC_*, and the surrounding medium (index) [13]. The coupling length, $L_{c}$, also known as the beating length, refers to the distance over which maximum energy is transferred between the coupled waveguides [14]. A shorter $L_{c}$ can be achieved providing the coupling is stronger (i.e., higher *k*), hence the ratio of the input/output power splitting as well as the degree of polarisation splitting, is a function of the material and device parameters [15,16]. Assuming negligible coupling between the S-bend waveguide sections, then the optical power of through and coupled ports (*P*_2_ and *P*_3_, respectively) can be described using coupled-mode theory, according to [17]:

(1) $$P_{2}=P_{1}{cos}^{2}\left(\frac{\pi L_{DC}}{2L_{c}}\right)$$

(2) $$P_{3}=P_{1}{sin}^{2}\left(\frac{\pi L_{DC}}{2L_{c}}\right)$$

where *L_DC_* is the length of the straight waveguide section of the DC, $L_{c}=\lambda /2\Delta n_{eff}$ in which $\Delta n_{eff}$ describes the difference of the effective indices between the even ($n_{eff\_even}$) and odd ($n_{eff\_odd}$) super-modes, and λ is the wavelength of transmitted light. The periodic pattern of the maximum output power, which depends on both *L_DC_* and $L_{c}$ (and therefore the effective index of the device), creates an opportunity to use the device as a refractive index sensor. The sensitivity of such a sensor is determined by the strength of the interaction between the evanescent wave that extends into the surrounding medium and the surface-bound target analyte. The mode confinement (or, in contrast, the penetration depth of the evanescent field into the low-index cladding) is determined by the refractive index contrast between the silicon waveguide core and the upper cladding, which in turn controls the effective indices of the even and odd modes of the DC (i.e., $\Delta n_{eff}$). The sensing modality is thus realised by exploiting the fact that the output spectrum is a function of both λ and $n_{eff}$.

## 2. Materials and Methods

**Figure 2:**
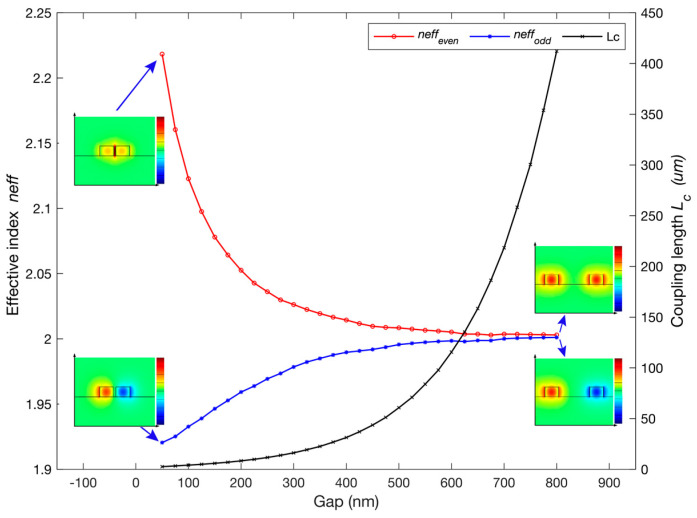
Effective refractive indices of the even ($n_{eff\_even}$) and odd ($n_{eff\_odd}$) super-modes and coupling length ($L_{c}$) as a function of the separation gap. The insets demonstrate the modes profiles for a DC with gap = 50 nm (**left**) and gap = 800 nm (**right**).

When light propagates in one arm of the DC, the amount of power that is either transmitted or coupled varies sinusoidally as a function of wavelength, $\Delta n_{eff}$ and *L_DC_*. The difference in effective indices between the even and odd modes ($\Delta n_{eff}$) of a DC can be controlled by several design parameters, including the waveguide dimensions, the refractive index contrast between the waveguide and cladding materials, and the gap size between the two waveguides. The width and height of the designed waveguides are w = 350 nm and h = 220 nm, respectively. The RI’s used in the model for the silicon waveguide core and buried oxide (BOX) are 3.48 and 1.44, respectively [18,19]. The device’s top cladding index is initially defined to be that of water, i.e., with RI = 1.318 at a wavelength of 1550 nm [20]. The gap size between the two waveguides in the DC can affect $\Delta n_{eff}$ and $L_{c}$ as shown in Figure 2. The red, blue and black curves correspond to $n_{eff\_even}$, $n_{eff\_odd}$ and $L_{c}$, respectively. The insets demonstrate the profile of the electric field distribution of the fundamental TE mode. The left and right insets show the profile for a waveguide separation gap of 50 nm and 800 nm, respectively.

A smaller gap size leads to a larger difference in effective indices and vice versa. However, the gap size also affects the coupling strength and hence the coupling length, $L_{c}$. In this paper, a gap of 50 nm was chosen to achieve high sensitivity in refractive index sensing with a small coupling length (for this device $L_{c}=$ 2.60 μm), hence more compact device dimensions. 

The distance between two consecutive positive peaks is the free spectral range (FSR), expressed as:

(3) $${FSR}_{DC}(\lambda )=\frac{\lambda^{2}}{L_{DC}\Delta n_{eff}}=\left(\frac{{2L}_{c}}{L_{DC}}\right)\lambda$$

The wavelength shift (${∆\lambda }_{DC}$), arising from a variation in the refractive index (RI), is determined by [21]:

(4) $${∆\lambda }_{DC}=\left(\frac{\partial n_{eff\_even}}{\partial RI}-\frac{{\partial n}_{eff\_odd}}{\partial RI}\right)\frac{\lambda_{0}}{\Delta n_{g0}}$$

where $\lambda_{0}$ is the unperturbed wavelength, i.e., in the absence of any change in RI, $\left(\frac{\partial n_{eff\_even}}{\partial RI}-\frac{{\partial n}_{eff\_odd}}{\partial RI}\right)$ represents the instantaneous change in super-mode effective indices with respect to a change in the surrounding RI and $\Delta n_{g0}$ denotes the group index variation between the even and odd super-modes of a reference DC (i.e., not subject to any external perturbations). The device sensitivity is determined from the shift in resonant wavelength in response to a change in RI, $S={∆\lambda }_{DC}/∆RI$.

**Figure 3:**
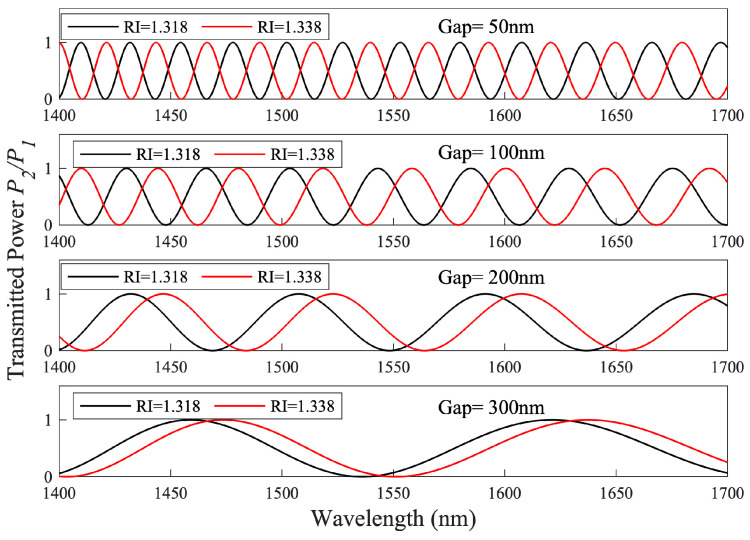
Spectra of the transmitted power, collected from *P*_2_ for a DC immersed in water with RI of 1.318 (black) and the response (red) to an increase in RI of 1.338, as a function of λ for different values of G.

Figure 3 represents the spectrum of the output power (*P*_2_/*P*_1_) as a function of wavelength. The shifted response (red curves) is the result of ΔRI = 0.02. As expected, in this case, the sensitivity S was found to be inversely proportional to the waveguide separation gap, G, with values for S = 86, 113, 159 and 177 nm/RIU determined for G = 300, 200, 100 and 50 nm, respectively. 

### 2.1. Single Directional Coupler-Assisted Racetrack Resonator (DCARR)

**Figure 4:**
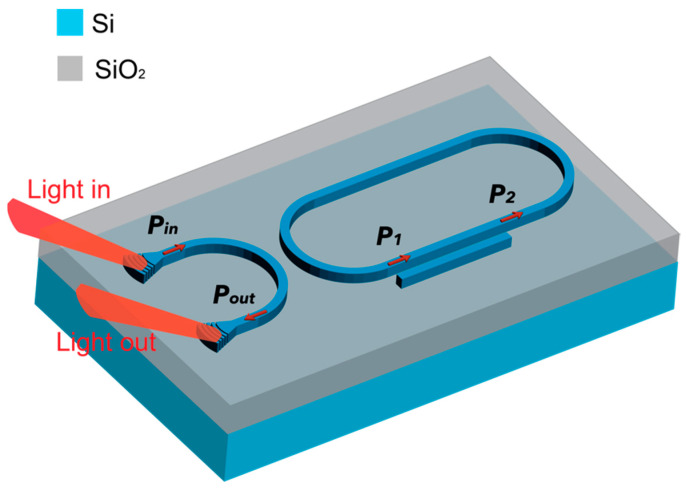
Schematic illustration of the proposed SOI-DCARR sensor, showing the inputs *P_in_* (*P*_1_) and outputs *P_out_* (*P*_2_) ports for RR (DC section).

In this paper, we begin by describing an approach whereby a single DC section is coupled to a racetrack-type resonator, forming what we refer to as the DC-assisted racetrack resonator (DCARR), Figure 4. This device exploits and combines the advantages of both the RR and DC, resulting in an ultra-large FSR compared with the traditional ring or racetrack resonators. Furthermore, the proposed device not only exploits the operating principle of the DC as a sensor, described earlier in [22], but also enhances it by integration with the RR resonant cavity [23].

Steglich et al. previously reported a design integrating a strip-to-slot waveguide mode-converter with a RR cavity with the primary objective of increasing the Q-factor in order to improve spectral sensitivity [24]. Our proposed device is significantly simpler and easier to fabricate as it does not include the complex mode-converter, which can be challenging to fabricate [25]. The DCARR device incorporates a single-point coupling section with an abruptly introduced slot waveguide (as the DC) section. 

**Figure 5:**
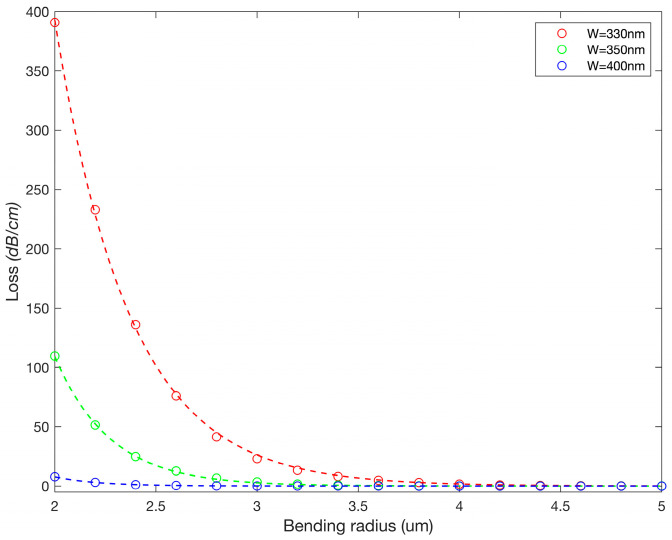
Simulated bending losses (circles) as a function of the SOI waveguide width and exponential fits (dashed lines).

A key feature of this design is the extension of FSR, which is facilitated by the DC segment. Various applications, including sensing and filters, require large FSR, but for a single RR, this can also be challenging as it necessitates a cavity with a small perimeter (i.e., tighter bend radius), which translates to larger optical losses, as depicted in Figure 5.

This has motivated the design of a range of novel structures that can extend the RR FSR whilst attempting to minimise bend losses. For example, Li et al. described a cavity that consists of a ring coupled to an asymmetric loop-MZI reflector with FSR = 150 nm [26]. An alternative approach is to couple multiple resonators of different sizes to produce an envelope signal (optical Vernier effect) for which only overlapping resonances are transmitted to a common output [8,27,28]. However, one drawback of these types of devices is their rather large footprint and relatively complex design that is highly susceptible to fabrication tolerance. 

#### Single DCARR–Modelling

**Figure 6:**
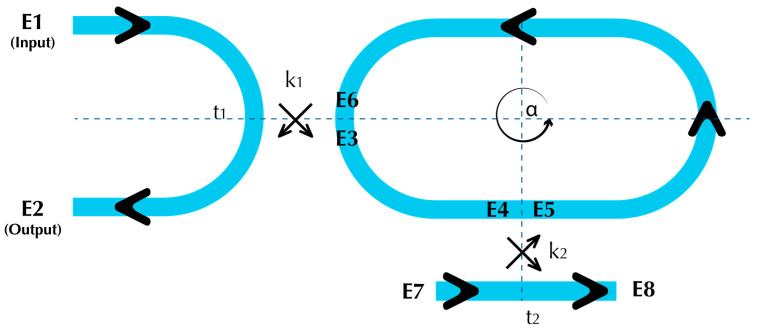
Schematic diagram of the single DCARR model, with input, output and transmitted fields, E1 to E8, coupling and transmission coefficients, *k*_1,2_ and *t*_1,2_ and the RR loss coefficient, α.

The single DCARR, shown in Figure 6, is modelled through analysis of the electric field in each section of the device. A single-point coupler is used to couple the input/output light (E1/E2) into and out of the RR cavity. The main advantage of employing this coupler type is to reduce the reliance of the transmission coefficient (*t*_1_) on wavelength [29]. As the coupled optical mode starts to circulate inside the cavity and eventually reaches the DC section, the field E4 will act as an input to the DC, while E5 will be significantly affected by the transmission coefficient (*t*_2_). In contrast to the single-point coupler, the long(er) coupling length induced by the adjacent waveguide (DC) results in a *t*_2_ that has a strong wavelength dependence [30]. In other words, the output transmission spectrum (E2) of a resonator that includes only a single-point coupler (*t*_1_, *k*_1_), in the absence of the DC (*t*_2_, *k*_2_), will simply be that of the RR, with resonances of nearly equal extinction ratio (ER). The inclusion of the DC section sinusoidally varies the magnitude of the RR resonances appearing at E2.

The DCARR can be modelled using the well-known matrix analysis of an RR [31]. The resonance frequencies or wavelengths, coupling and transmission coefficients, and transmission spectra of the resonator are calculated by relating the output and input fields according to:

(5) $$\left(\begin{array}{c} E2\\ E3 \end{array}\right)=\left(\begin{array}{cc} t_{1} & i\kappa_{1}\\ i\kappa_{1} & t_{1} \end{array}\right)\left(\begin{array}{c} E1\\ E6 \end{array}\right)$$

(6) $$\left(\begin{array}{c} E5\\ E8 \end{array}\right)=\left(\begin{array}{cc} t_{2} & i\kappa_{2}\\ i\kappa_{2} & t_{2} \end{array}\right)\left(\begin{array}{c} E4\\ E7 \end{array}\right)$$

(7) E4E6=α14eiθ400α34ei3θ4E3E5

The single-point coupling is controlled by the coefficients *t*_1_ and *k*_1,_ and the DC is characterised by *t*_2_ and *k*_2_. The overall cavity losses (i.e., absorption, scattering, and radiation) are incorporated in the loss coefficient, α, where α=1 indicates a lossless internal cavity. In Equation (7), the subscript associated with the loss coefficients (i.e., 14 and 34) represent the fraction of the roundtrip loss coefficients. Similarly, the accumulated phase, θ is multiplied by the same factors to evaluate the phases of E4 and E6 after quarter and three-quarter roundtrips, respectively [31]. The accumulated phase, θ, around the entire cavity can be expressed as:

(8) $$\theta =\frac{2\pi L{\bar{n}}_{eff}}{\lambda }$$

where *L* is the cavity roundtrip length (perimeter) and ${\bar{n}}_{eff}$ represents the weighted average of the effective indices of the radial, straight and DC sections of the RR, as determined from [24]:

(9) $${\bar{n}}_{eff}=\left(\frac{L_{DC}}{L}\right)n_{effs}+\left(\frac{2\pi R}{L}\right)n_{effb}+\left(\frac{L_{DC}}{L}\right)n_{eff(DC)}$$

where *R* is the radius of the curved sections of the cavity, $n_{effs}$ and $n_{effb}$ denote the effective indices of the straight and curved waveguide sections, respectively and $n_{eff(DC)}$ is the difference between the effective index of the individual straight waveguide and the mean value of the even ($n_{even}$) and odd ($n_{odd}$) super-modes, expressed as [32]:

(10) $$n_{eff(DC)}=n_{effs}-\left(\frac{n_{even}+n_{odd}}{2}\right)$$

All of the effective indices were obtained using the Finite Difference Eigenmode (FDE) method. 

Assuming the input light magnitude E1 = 1, no external light is injected into the DC (i.e., E7 = 0) and a lossless, single-point coupler (i.e., ${t_{1}}^{2}+{k_{1}}^{2}=1$), then the output power (*P_out_*) can be determined by solving Equations (5)–(7):

(11) Pout=E2E12=t2eiθα14α34−t1t1t2eiθα14α34−12

The physical length of the DC (*L_DC_*) and the coupling length (*L_c_*) have a significant impact on the transmission coefficient *t*_2_, which is given by [30,32]:

(12) $$t_{2}=cos\left(\frac{\pi L_{DC}}{2L_{c}}\right)e^{i\left(\frac{2\pi n_{eff(DC)}}{\lambda }\right)L_{DC}}$$

**Figure 7:**
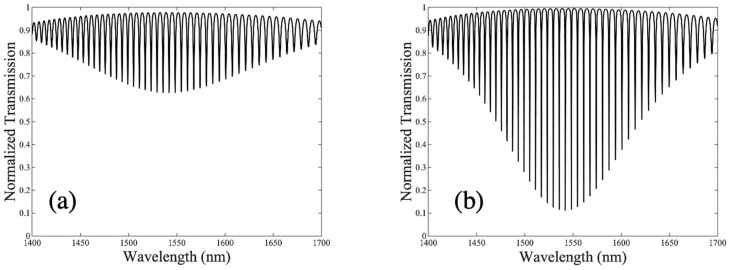
Transmission spectra ($P_{out}$) for a single DCARR with *L_DC_* = 20.7 μm and *R* = 10 μm, $t_{1}$ = 0.95 and (**a**) α = 0.80 (**b**) α = 0.95.

By integrating the DC in this configuration, it becomes possible to significantly extend the FSR of the isolated RR. The interference of the circulating mode in the cavity and the light coupled from the DC allows a periodic envelope transmission spectrum ($P_{out}$) comprising constituent peaks (resonances) where the FSR of the transmitted envelope is the same as the DC FSR illustrated by Equation (3). Figure 7 illustrates an ultra-large FSR for a DCARR with *R* = 10 μm and *L_DC_* = 20.7 μm. The mean group index (${\bar{n}}_{g}$) is used instead of ${\bar{n}}_{eff}$ in the modeling of the DCARR (i.e., in Equation (8)) because it takes into account the effect of dispersion on the propagation of light in the resonator and can be written as [24,32]

(13) $${\bar{n}}_{g}={\bar{n}}_{eff}-\lambda \left(\frac{\partial {\bar{n}}_{eff}}{\partial \lambda }\right)$$

Figure 7 compares the effect of cavity loss on the envelope with (a) $t_{1}$ = 0.95 and α = 0.80 and (b) α = $t_{1}$ = 0.95 (i.e., critical coupling condition), illustrating, for the latter, a larger ER and narrower full width at half maximum (FWHM) of the envelope. The optical phase shift of the DC can be employed to derive the resonance shift of the envelope (${∆\lambda }_{env}$) that occurs due to a change in the cover RI, resulting in:

(14) $${∆\lambda }_{env}={∆\lambda }_{DC}$$

For this device, S can be as high as 183 nm/RIU with an envelope FSR = 390 nm, for λ = 1550 nm. These values can be enhanced further still by optimisation of the DC length or by implementing Vernier cascaded DCs, as discussed in the next section.

### 2.2. Novel Double Directional Coupler-Assisted Racetrack Resonator (DCARR)

**Figure 8:**
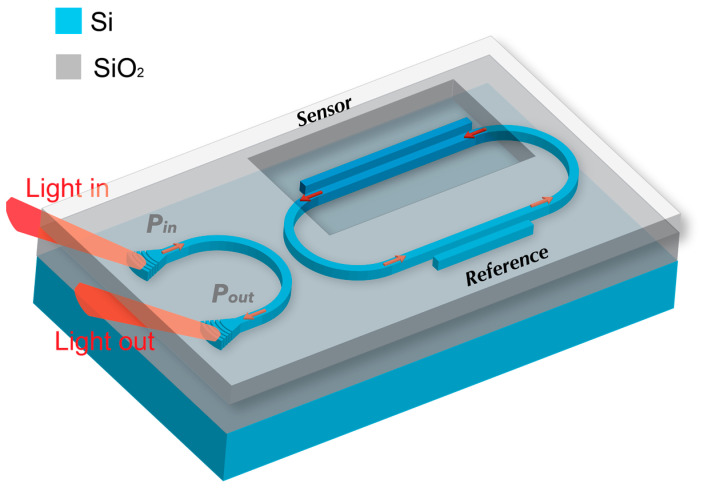
Schematic illustration of the double DCARR with silica-clad reference and exposed sensor DCs, on either side of the racetrack resonator cavity.

By extending the single DCARR to a double DCARR, we can exploit the optical Vernier effect. In this innovative design, the first DC is positioned beneath a SiO_2_ top cladding and acts as a reference, while the second is exposed to the surrounding environment and serves as the sensor. The exposed DC can detect changes in RI of the near optical field, as demonstrated in Figure 8. The novelty of the proposed device lies in its configuration and the significantly improved sensitivity and FSR.

For the light of different wavelengths coupled into this device, the output transmission spectrum ($P_{out}$) is strongly affected by the transmission coefficients associated with the single-point coupler, reference and sensor DC. Therefore, the optical Vernier effect is obtained by ensuring that the reference and sensor DCs have different transmission coefficients. This can be achieved by creating a difference in the separation gap (G) or *L_DC_* between the two DCs.

#### Double DCARR–Modelling

**Figure 9:**
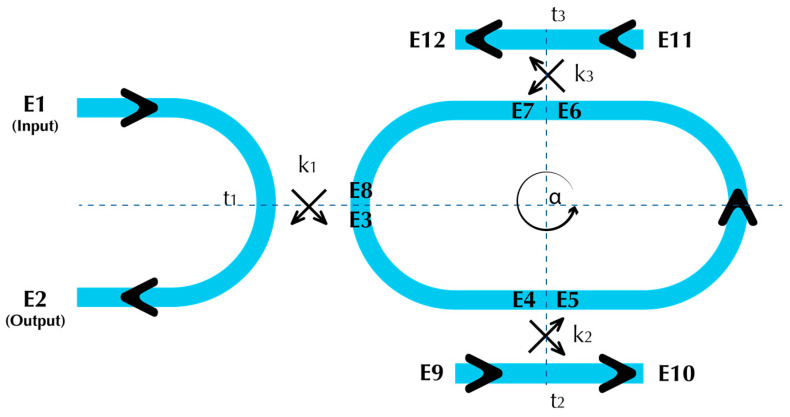
Double DCARR configuration showing the electric fields, coupling and transmission coefficients in each section of the model.

A schematic diagram of the dual DCARR model is illustrated in Figure 9.

Analogous to the single DCARR, the normalised transmitted power ($P_{out}$) is obtained analytically by solving the following four matrices:

(15) $$\left(\begin{array}{c} E2\\ E3 \end{array}\right)=\left(\begin{array}{cc} t_{1} & i\kappa_{1}\\ i\kappa_{1} & t_{1} \end{array}\right)\left(\begin{array}{c} E1\\ E8 \end{array}\right)$$

(16) $$\left(\begin{array}{c} E5\\ E10 \end{array}\right)=\left(\begin{array}{cc} t_{2} & i\kappa_{2}\\ i\kappa_{2} & t_{2} \end{array}\right)\left(\begin{array}{c} E4\\ E9 \end{array}\right)$$

(17) $$\left(\begin{array}{c} E7\\ E12 \end{array}\right)=\left(\begin{array}{cc} t_{3} & i\kappa_{3}\\ i\kappa_{3} & t_{3} \end{array}\right)\left(\begin{array}{c} E6\\ E11 \end{array}\right)$$

(18) E4E6E8=α14eiθ4000α12eiθ2000α14eiθ4E3E5E7

Assuming, for simplicity, that the reflected waves at E10 and E12 are neglected, the normalised output power as a function of the transmission coefficients, loss factor and accumulated phase, θ can be written as:

(19) Pout=E2E12=t2t3eiθα12α142−t1t1t2t3eiθα12α142−12

**Figure 10:**
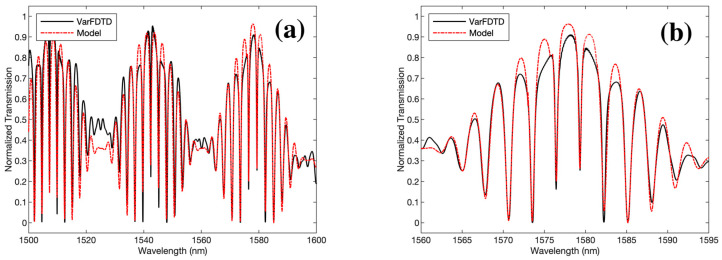
The normalised output spectrum using varFDTD (black curve) and our matrix-based model (red dashed curve) across (**a**) the entire wavelength range (1500–1600 nm) and (**b**) for a single, 35 nm (1560–1595 nm) envelope.

The transmission coefficients *t*_1_, *t*_2_ and *t*_3_ represent the single-point coupler, reference and sensor DCs, respectively. Using the following parameters in our model; *R* = 3.1 μm, $L_{{DC}_{ref}}$= $L_{{DC}_{sensor}}$ = 100 μm, the normalised transmission spectrum of the device is depicted in Figure 10, along with a comparison using the Lumerical variational Finite-Difference Time-Domain (varFDTD) solver, which accounts for the effects of reflection, transmission, and scattering of electromagnetic waves at interfaces and boundaries. The very good agreement between the analytical and simulated spectra indicates that the neglection of the reflected waves is an acceptable assumption. Therefore, the proposed model provides a simple, fast and more easily customisable method as compared to the FDTD (or varFDTD), which is slow and more computationally expensive.

For this type of device, the improvement in FSR is determined by a factor known as the Vernier coefficient (*V*), given by:

(20) $$V=\frac{{FSR}_{ref}}{{FSR}_{ref}-{FSR}_{sensor}}$$

where ${FSR}_{ref}$ and ${FSR}_{sensor}$ denote the individual FSRs of the reference and sensor DCs. The wavelength shift of the output envelope (${∆\lambda }_{Vernier}$) and sensitivity ($S_{Vernier}$) resulting from a change, ΔRI, are given by:

(21) $${∆\lambda }_{Vernier}=V{\cdot ∆\lambda }_{sensor}$$

(22) $$S_{Vernier}=\frac{{∆\lambda }_{Vernier}}{∆\mathrm{RI}}$$

where ${∆\lambda }_{sensor}$ represents the wavelength shift due to a change in the surrounding RI of the exposed sensor DC. In a design that features a large value of *V*, the $S_{Vernier}$ can be exceptionally high. However, while a large value of *V* is generally desirable, it can cause the transmission envelope to shift outside of the operating range of the device or optical detection instrumentation. Therefore, to optimise the practical performance of the device, it is necessary to carefully balance the desired $S_{Vernier}$ with the limitations of the detection instrumentation, taking into account the trade-off between sensitivity and dynamic range. Assuming negligible dispersion, the FSR of the envelope (${FSR}_{Vernier}$) can be expressed as [27]:

(23) $${FSR}_{Vernier}=\frac{{FSR}_{ref}\cdot {FSR}_{sensor}}{\left|{FSR}_{ref}-{FSR}_{sensor}\right|}$$

## 3. Results and Discussion

The proposed double DCARR provides many advantages over traditional optical ring resonators, as it provides an extremely high bulk sensitivity and FSR, allowing it to be used in sensing or optical filter applications. This novel device has a waveguide width of w = 350 nm and a height of h = 220 nm. In addition, the separation gap, G, of the single-point coupler and the integrated DCs are 100 nm and 50 nm, respectively. Hence, it is compatible with standard UV lithographic processing and standard 220 nm SOI technology.

**Figure 11:**
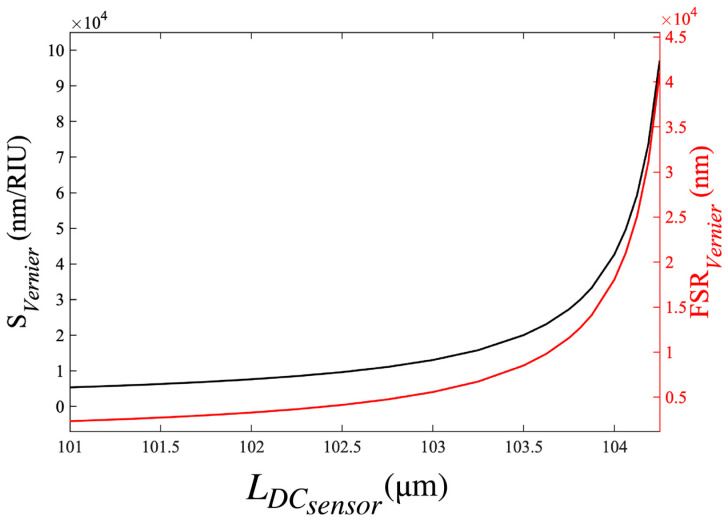
The double DCARR sensitivity, $S_{Vernier}$, (black curve) and envelope ${FSR}_{Vernier}$ (red curve) as a function of the length of the sensor DC, $L_{{DC}_{sensor}}$.

Figure 11 shows $S_{Vernier}$ (black curve) and ${FSR}_{Vernier}$ (red curve) calculated from Equations (22) and (23) as a function of $L_{{DC}_{sensor}}$ for the double DCARR, with a fixed $L_{{DC}_{ref}}=$ 100 μm and a change in the cover index, ΔRI = 10^−3^. 

**Figure 12:**
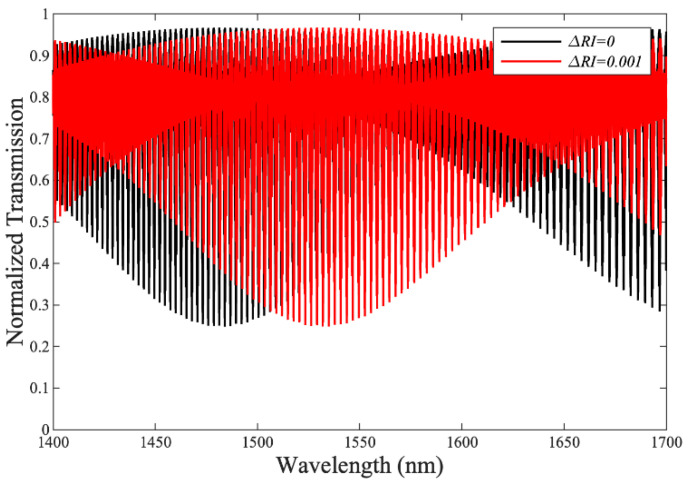
Unperturbed (black) and shifted (red) (for ΔRI = 10^−3^) envelope transmission spectra for a double DCARR device, with $L_{{DC}_{ref}}=$ 2590 μm and $L_{{DC}_{sensor}}=$ 2740 μm.

Although the theoretical $S_{Vernier}$ for this device can reach exceptionally large values (10^4^ to 10^5^ nm/RIU), this is concomitant with an exceptionally large FSR*_vernier_* ≥ 2000 nm, i.e., far exceeding the operating wavelength (1400–1700 nm) of such devices. Such a large envelope FSR would limit the practical use of the proposed device as a sensor (because it would be impossible to determine the exact wavelength shift of the envelope, even for very small changes in RI). This can be resolved by dramatically increasing the length of both reference and sensor DCs, although, of course, this implies a trade-off with device footprint. Optimised values for sensitivity with a practical FSR can be achieved by careful design of the DC lengths; for example, a device with $L_{{DC}_{ref}}=$ 2590 μm and $L_{{DC}_{sensor}}=$ 2740 μm exhibits ${FSR}_{Vernier}=$ 246 nm (within the operating wavelength range of the device). Figure 12 shows the unperturbed and the shifted envelope transmission spectra for this device for a small change ΔRI = 10^−3^. 

In this case, the exposed DC (sensor) has a top cladding index corresponding to that of water at 1550 nm, i.e., RI = 1.318 (and the reference DC is SiO_2_ clad), producing an envelope with a centre resonance (dip) at 1482 nm. As the environmental RI is increased slightly, by 10^−3^, the central envelope resonance shifts by 50 nm, which implies a very high $S_{Vernier}$ = 50,000 nm/RIU for a practical device operating in the 1400–1700 nm range. The sensitivity and FSR of the proposed device are compared with the previously reported refractive index-based sensors. Table 1 shows a comparison that includes SOI-based waveguide structures with their sensitivity measured in (nm/RIU) and FSR measured in (nm).

## 4. Conclusions

We numerically and analytically examined the coupling of resonant light in a racetrack-type cavity with single- and double (asymmetric)-DCs, as a novel approach to enhance refractive index-based sensing in chip-scale silicon PICs. In particular, for a double DCARR incorporating a (SiO_2_ clad) reference and (exposed) sensor DC, with carefully designed lengths, the optical Vernier effect is exploited to enhance both FSR and sensitivity. The results of our analytical modelling of the device(s) described here are in very good agreement with those derived from FDTD simulations. For the sample design of double DCARR proposed here, which has *R* = 10 μm, $L_{{DC}_{ref}}=$ 2590 μm and $L_{{DC}_{sensor}}=$ 2740 μm, an extremely high sensitivity, *S* = 5 × 10^4^ nm/RIU is achieved with a practical envelope FSR*_Vernier_* = 246 nm, for a RI-based sensor operating in the near-IR (1400–1700 nm) wavelength range. The proposed device, with its relatively small footprint, high sensitivity and large FSR, can have a significant impact on a variety of applications, including biomedical sensors, food safety, the chemical industry and environmental monitoring, where precise and accurate measurements of refractive index are needed in on-chip optical systems.

## Figures and Tables

**Table 1:** A comparison of waveguide-based refractive index sensors.

Structure Type	Sensitivity (nm/RIU)	FSR (nm)	Ref.
Double slot MZI	700	7	[33]
Sub-wavelength grating racetrack microring resonator	7061	179.89	[34]
MZI-ring	21,500	~18	[35]
TM mode based on cascaded double-ring resonators	24,300	105	[35]
DCARR	50,000	246	This work

## Data Availability

The data in this research can be made available at a reasonable request.
